# Envisioning grassroots science: DIY biology in Canada, Britain, and Germany

**DOI:** 10.1371/journal.pone.0334484

**Published:** 2025-12-09

**Authors:** Anna Verena Eireiner

**Affiliations:** Department of Sociology, University of Cambridge, Cambridge, United Kingdom; University of Gothenburg School of Business Economics and Law: Goteborgs universitet Handelshogskolan, SWEDEN

## Abstract

This study employs the conceptual lens of sociotechnical imaginaries to examine how DIY biology is envisioned in Canada, Great Britain, and Germany. DIY biology, an extra-institutional movement, aspires to democratize biology by making research infrastructures accessible, fostering open-source knowledge, and challenging the expert-public divide. Based primarily on 23 in-depth interviews with practitioners from three countries, complemented by insights from a global online survey of 152 DIY biologists and additional perspectives from policymakers, this research highlights how country-specific sociopolitical contexts shape the local identities and practices of a global movement. The findings reveal country-specific differences in the ways DIY biology is envisioned and materialized. Canadian DIY biologists often adopt entrepreneurial narratives akin to “garage start-ups,” critiquing the high cost of higher education and the rigidity of traditional research institutions. German practitioners, emphasizing sustainability and responsibility, critique capitalism while aligning their DIY efforts with environmental activism. In Great Britain, DIY biologists often focus on collective benefits, thereby highlighting global equity and collaboration. Despite their differences, DIY biologists across all three countries share aspirations to foster inclusivity, intellectual freedom, and socially relevant research, while dealing with challenges like a lack of funding to conduct their experiments and maintain laboratory spaces. By exploring how grassroots science movements navigate challenges and reimagine scientific practices, this study contributes to scholarship in Science and Technology Studies (STS). It provides an empirically rich, comparative perspective on how a global movement is envisioned within different sociopolitical contexts across countries. This research underscores the potential of DIY biology to democratize science but also calls for critical reflection on its systemic challenges, offering actionable insights for cultivating sustainable, community-driven scientific practices.

## 1. Introduction: Imagining DIY Biology in Canada, Great Britain, and Germany

Do-It-Yourself (DIY) biology, often referred to as biohacking or community biology, is a grassroots movement enabling individuals to conduct biological research outside traditional institutional settings [1,2]. DIY biology can be conceptualized under the umbrella of “extra-institutional science“, capturing professionalized communities and initiatives that create scientific knowledge outside of traditional academic, industry, or military research institutions. Extra-institutional science movements craft alternative epistemic spaces that challenge the boundary between science and publics, as well as remake the hierarchies, authorities, funding regimes, oversight structures, practices, and proprietary regimes of traditional spaces of scientific research [3]. Participants establish their laboratories in unconventional spaces such as garages or community hubs, sourcing equipment and materials primarily through online platforms [4]. The DIY biology movement is believed to have originated in the United States during the early 2000s, particularly in urban centers in the United States like Boston, San Francisco, and New York [5,6]. These cities, known for their vibrant innovation ecosystems, became host to communities of both professional and amateur scientists dedicated to democratizing biotechnology and pushing the boundaries of DIY experimentation.

DIY biology, as an internet-native movement, operates globally through digital platforms that share knowledge and connect practitioners [7,8]. However, despite its digital infrastructures and global research, the practices, values, and organizational forms of DIY biology are deeply shaped by the national and local contexts in which the movement’s practitioners operate. This paper details how DIY biologists broadly envision their movement and how these global envisionings are co-produced within three different country contexts. Moreover, this paper investigates the material results, which show how imaginaries get bound up with on-the-ground social and political realities. This is to say that this research identifies the movement’s three principal sociotechnical imaginaries to then show how DIY biologists in Canada, Great Britain, and Germany invoke and materialize these imaginaries in culturally unique and country-specific ways.

Canada, Great Britain, and Germany were selected as comparative cases not merely for their active communities but for the distinct institutional, cultural, and political contexts they offer. Each provides a different lens on how grassroots science is enabled, constrained, or redirected by local conditions. Canadian communities are diverse, ranging from well-established spaces like Vancouver’s Open Science Network to pop-up labs and Victoria’s Makerspace that also hosts a DIY biology laboratory [9–11]. In Great Britain, DIY biology has declined since its peak around 2016–2017, but still hosts a few active spaces, including London’s Biohackspace and Scotland’s ASCUS Lab, which merges art and science [12,13]. Germany hosts a comparatively vibrant and interconnected scene, with notable spaces like Top Lab in Berlin and Lab 3 in Dortmund, alongside a significant reliance on online platforms to sustain community organizing [14,15]. Rather than juxtaposing these cases descriptively, the comparison enables an analysis of how globally shared imaginaries of science and participation are received, negotiated, and uniquely transformed and embedded in country-specific contexts.

Theoretically, this paper is situated in culturally sensitive innovation studies, which emphasize the ways global visions of innovation are reinterpreted through context-based histories, policies, and cultures [16]. This perspective moves beyond a diffusion model to highlight the friction and processes that occur when global movements meet unique cultural contexts.

Methodically, this research draws on the 2021 DIY Biology Community Survey, a global survey aimed at identifying the shared sociotechnical imaginaries that underpin the global DIY biology movement. Moreover, I conducted 28 in-depth interviews with DIY biologists and relevant regulators in Canada, Great Britain, and Germany, exploring how these global visions are co-produced within specific national contexts shaped by distinct sociopolitical circumstances.

By combining these data sources, this research operationalizes the relationship between the digital infrastructure of DIY biology and its national grounding, offering a framework to understand how global movements materialize differently across institutional contexts.

This work contributes to the existing literature by showing how sociotechnical imaginaries of DIY biology are interpreted and adapted within distinct national contexts. While previous studies have explored the general characteristics and aspirations of DIY biology as a global movement [1,4,17], few have analyzed how these broad visions are co-produced and reshaped through interactions with local cultural, regulatory, and political environments. By focusing on Canada, Great Britain, and Germany, this research highlights the localized nature of DIY biology and reveals how global ideals intersect with country-specific narratives, thereby enriching our understanding of the multifaceted nature of grassroots scientific communities.

Recognizing that DIY biology operates as both a global and local movement, I approached the study through the lens of culturally sensitive innovation studies. This perspective challenges the assumption that innovation simply crosses borders to be adapted more or less seamlessly, emphasizing instead the nuanced ways in which global ideas interact with local specificities [16].

In the first part of this paper, I introduce the conceptual lens of sociotechnical imaginaries [18] and outline the methods employed.

In the second part of this paper, I introduce three sociotechnical imaginaries that emerged through a review of relevant literature and a thematic analysis of survey and interview data. First, DIY biologists commonly imagine their movement as an avenue that allows for the exploration of socially relevant research agendas. Second, DIY biology is envisioned to democratize the scientific enterprise. Third, DIY biologists argue that extra-institutional science allows for greater intellectual freedom than traditional academic research. These three globally shared sociotechnical imaginaries guide my investigation into how DIY biology is imagined in country-specific contexts, shedding light on the fundamental tensions brought about by the boundary-challenging activities of DIY biologists.

The third part of this paper is dedicated to the three countries under investigation. For each country, I follow the same structure. I first give an overview of the regulatory country context in which DIY biologists build their spaces and communities. I then introduce country-specific imaginaries through the lens of the three core imaginaries detailed earlier. I then focus on how DIY biologists materialize their communities and spaces and the challenges they encounter in the process. In this context, “materializing” refers to DIY biologists’ efforts in putting their visions of extra-institutional science into existence, both in material and immaterial ways. This is followed by a concluding section that synthesizes this paper’s findings.

## 2. Methods

### 2.1. Sociotechnical imaginaries and materializing DIY biology

This project contributes to a context-specific notion of innovation. It draws on the co-productionist strand of Science and Technology Studies (STS) and, specifically, the concept of “sociotechnical imaginaries” to theorize the country-specific engagement with the DIY biology movement. Innovations in science and technology, as well as concomitant uncertainties, are one of the prime examples where sociotechnical imaginaries are invoked and challenged. Sociotechnical imaginaries are defined as “collectively held, institutionally stabilized, and publicly performed visions of desirable futures” that are “animated by shared understandings of forms of social life and social order, attainable through, and supportive of, advances in science and technology” [19: 4]. How the future of, and with, DIY biology is imagined is not grounded in pure speculation. Promises for the future influence current actions [20]. The processes of deliberation in all three countries show that expectations mobilize actors. The ways that DIY biology is envisioned in the three countries of investigation are the result of processes that are deeply ingrained with social, economic, cultural, and political aspects and historical contingencies. STS can make implicit imaginaries, their framings and agencies, explicit, to open up pathways and impact social, political, and technical choices [19].

Hence, analytically, I put the tension between the supposedly universal mechanics of a globally networked DIY biology movement and its innovative DIY practices, and the unique country-specific sociocultural contexts of implementation, front and center. In each country, I trace the envisionings of national DIY biology communities to show how implementations of the seemingly “same” movement lead to different interpretations and outcomes as they align with country-specific visions, rationales, and identities.

To complement this focus on sociotechnical imaginaries, I attend to how such visions materialize in the practical work of DIY biology. STS has shown that future-oriented imaginaries are not only discursive but also enacted through infrastructures, practices, and situated forms of expertise [21,22]. Building on STS scholarship that highlights how non-traditional actors contribute to the shaping of scientific knowledge and infrastructures, I consider DIY biologists not as simple technology users but as practitioners actively producing and negotiating the socio-material conditions of science outside institutional settings [23,24]. DIY biology puts forth a rethinking of scientific organizations and commitments. Its members, explicitly or implicitly, introduce a distinct set of values that are enacted in the spaces and practices they craft. This, of course, is not without challenges, frictions, and tensions. DIY biology provides us with a site to investigate how alternative science is imagined and put into practice. This attention to material practices, with their alignments, tensions, and frictions with envisioning, resonates with work on the performativity of sociotechnical imaginaries [25,26]. This research highlights how visions of science are shaped, challenged, and redefined through the on-the-ground realities of making DIY biology communities.

### 2.2. Survey and personal interviews

The following insights are based on existing scholarly literature, findings from my 2021 DIY Biology Community Survey [27], and data gathered through 23 semi-structured interviews with DIY biologists and regulators in charge of overseeing DIY biology activities. This research has been approved by the Ethics Committee at the University of Cambridge, Department of Sociology in January 2020.

Between May 2020 and September 2022, I conducted interviews with DIY biologists (23 participants) and regulators in charge of overseeing DIY biology activities in the three countries (5 participants).

I selected DIY biology interviewees by virtually attending community networking events (e.g., the Global Community Biosummit 2020 and 2021, [28]). I also recruited participants in person at DIY biology community labs and events in Great Britain, Germany, and Canada. This recruitment process may have introduced a selection bias, likely favoring extroverted, well-connected individuals active in international contexts and the countries I visited.

Potential interviewees were provided with a brief introduction to the project. Those who agreed to participate signed an informed consent form prior to the interview.

DIY biology participants ranged in age from their early twenties to late fifties, with a significant concentration in their late twenties and early thirties. The sample showed an almost equal gender distribution, and the majority of participants were highly educated, holding advanced degrees in the natural sciences. These demographics closely align with existing data on the DIY biology community [29].

I chose semi-structured interviews to allow for consistency in posing open-ended questions while maintaining the flexibility to follow up on emerging topics of interest [30, p. 319, 31]. While the core questions remained the same, their order varied, and participants were encouraged to elaborate on their experiences ([31], p. 53). Conducting research during the COVID-19 pandemic introduced challenges to data collection, limiting some in-person interactions. Additionally, interviews conducted virtually may have influenced participant openness and the overall interview dynamics compared to face-to-face settings.

The interviews were conducted either in person or remotely via video chat, with audio recordings made only after obtaining prior consent. The questions addressed DIY biologists’ motivations, challenges, and backgrounds in academia and industry, as well as their interactions with regulators and sponsors.

Interview recordings were transcribed with the help of Descript transcription software. I then analyzed the transcripts using NVivo software. The qualitative data was analyzed using Braun and Clarke’s [32,33] reflexive thematic analysis. This was done following six recursive phases: (1) familiarization with the data, (2) generation of initial codes, (3) searching for themes, (4) reviewing themes, (5) defining and naming themes, and (6) producing an analysis. Codes were refined through several iterative rounds as themes were compared across national contexts and adjusted to reflect the analytic focus on sociotechnical imaginaries.

While this study follows Braun and Clarke’s steps, it also places emphasis on the aspect of reflexiveness in their approach, which, as Clark and Braun [33] point out, differs from traditional thematic analysis. Rather than treating themes as naturally emerging from data, this approach acknowledges that the researcher’s perspective actively shapes the analysis. In contrast to more positivist forms of thematic analysis that strive for neutrality or objectivity, reflexive thematic analysis intentionally acknowledges and weaves in the researcher’s interpretive standpoint, making subjectivity an integral and valuable part of meaning-making.

Throughout the analysis, I engaged in reflexive memo writing in fieldwork journals to trace the development of codes and themes and to ensure transparency of interpretive decisions. This process aimed to maintain consistency while allowing flexibility for emergent insights.

To protect participants’ identities, personal information was anonymized by removing identifiable details and replacing them with generic descriptors (e.g., “Community Member, Interviewee #8”). Participants were informed of this anonymization procedure as part of the informed consent process.

The 2021 DIY Biology Community Survey, a central element of this research, was active between March and November 2021. It included 28 questions with various response formats and was conducted using Qualtrics survey software. The survey was distributed across platforms such as Twitter, Facebook, Slack, DIY biology-specific email lists, and meet-up groups. Out of 154 respondents, 152 completed multiple questions, contributing to the final dataset. While the survey is not fully representative of the global DIY biology community, of which the DIYbio Google Group alone had over 5,100 members as of 2022, it provides valuable insights into the community’s landscape. Notably, it explores themes that receive less attention, such as DIY biology’s ties to academia and industry. Participation was open to all self-identified DIY biologists, ensuring diverse perspectives were captured.

Survey respondents were presented with an informed consent statement detailing the study’s purpose, duration, and any associated risks. Survey data was filtered, cleaned, classified, and merged in Qualtrics, the themes developed during interview analysis were used as an interpretive frame for the survey responses, enabling comparison across data sources.

During interviews, I began with initial questions but followed up to clarify or encourage participants to elaborate on their experiences in practicing and developing DIY biology communities within their specific countries. This data primarily informs the discussion of how DIY biology is envisioned and materialized in local contexts.

To provide context on the regulatory environments DIY biologists encounter in each country, I interviewed and corresponded via telephone and email with representatives from the primary institutions responsible for overseeing DIY biology, namely, the Public Health Agency of Canada (PHAC), the Health and Safety Executive (HSE) in Great Britain, and, in the case of Germany, regulatory stakeholders at the state level, given that oversight in this area is handled by the individual states rather than a single federal authority. In addition, I carried out a content analysis of relevant governmental publications on DIY biology in each country to examine how these communities are framed within national science and biotechnology governance. I collected policy documents through keyword searches on governmental websites and databases, supplemented by materials shared by policymakers. My coding combined deductive and inductive approaches: I started with predefined categories (e.g., regulatory actions, historical framings, sociotechnical imaginaries, perceived risks/benefits, portrayals of expertise), then iteratively refined and expanded them through open coding [34].

This chapter now turns to the findings, showing how DIY biology is imagined, practiced, and challenged across the three country settings studied.

### 2.3. Limitations

This study has several limitations. The qualitative analysis was conducted by a single researcher, which may have influenced data interpretation through individual positionality and subjectivity. While steps such as reflexive memo writing and iterative coding were undertaken to enhance transparency and rigor, intercoder reliability was not established. The absence of multiple coders may limit the validation of identified themes and reduce the robustness of claims. Additionally, recruitment methods, particularly the reliance on community events and online forums, may have led to a sample skewed toward highly networked participants. DIY biologists engaged in individual practices or working in home laboratories may be underrepresented. These limitations should be considered when interpreting the findings, which nevertheless provide valuable insights into how DIY biology communities envision and enact alternative forms of scientific practice.

## 3. Results

### 3.1. Envisioning a global movement: Three core imaginaries

This section uses data from personal interviews and the 2021 DIY Biology Survey to distill three sociotechnical imaginaries that define the movement as envisioned by its global practitioners. To enrich and contextualize these findings, it integrates scholarly accounts of DIY biology, offering further evidence of how the movement is conceptualized as a global phenomenon. In the next section, these three globally shared sociotechnical imaginaries are analyzed within the frameworks of three specific country contexts, illustrating how a global movement is co-produced through the lens of each nation’s distinct social, political, and historical traditions.

In personal interviews and my 2021 DIY Biology Community Survey, participants generally imagined their movement along the lines of three themes: socially relevant research agendas, the democratization of science, and intellectual freedoms. These large-scale visions are collectively held by DIY biologists across country contexts, which is why I identify them as sociotechnical imaginaries (see Jasanoff & Kim, 2009).

These three sociotechnical imaginaries emerged through in-depth thematic analysis of both interview and survey data following the approach laid out by Braun & Clarke as I detailed earlier [33]. These recurring, cross-cutting sociotechnical imaginaries offer a useful tool to better understand DIY biology’s motivations in the construction of alternative epistemic spaces. In the following, I describe and situate each of these sociotechnical imaginaries within existing literature, describing how these imaginaries are thought to run counter to established research institutions in industry and academia. Additionally, I point out potential conflicts and pitfalls that arise in the identity construction of the movement.

#### 3.1.1. Democratization of science.

The first sociotechnical imaginary is the democratization of biotechnology. Frow observes that the calls for “democratization” by DIY biologists are threefold: a call towards accessibility of research infrastructures, a challenge to the expert-publics divide, and the formation of open-source ownership regimes [17].

2021 DIY Biology Community Survey participants voice that they feel strongly about all of these aspects. Many participants specifically voice that they favor and want to advance DIY biology’s non-proprietary knowledge regime. This alternative knowledge regime is thought to support the goal of socially relevant research. Instead of patenting and/or monetizing new technologies, DIY biologists seek to disseminate their findings in hopes of collective benefit. This, again, is thought to counter the neoliberal academy. In the neoliberal academy, knowledge is not freely disseminated but patented in hopes of generating new market opportunities for capitalist endeavors.

DIY biologists also advocate for accessible research infrastructures and civic engagement and education, which is thought to counter elitist structures, academic paternalism, and knowledge privatization [1]. Research participants voice that they seek to make hands-on science education accessible to a wider audience. One 2021 DIY Biology Community Survey participant explains how DIY biology is an empowering experience as compared to traditional science education:

“A lot of biology is poorly taught, and I think a lot of people do biology because it was something that was always denied to them in their education. I like it because I get to feel and do the very thing I studied and it’s totally different from what they teach you in school. Oftentimes, there’s only so much you can learn in diagrams and lectures; you really do have to do it yourself to get a feel for the feedback it takes to do real biology. And it’s even better when you get to reconstruct things yourself and you get an appreciation for how much work was done in this world.”

Unlike universities, spaces of extra-institutional science may provide an open and inviting and, thus, accessible environment. Scholars observe that “in contrast to academic laboratories, the places where DIYbio is carried out usually allow access to everyone, regardless of their academic and socio-cultural background” [5]. It is often claimed that DIY biology enables new forms of civic engagement in contemporary knowledge societies that not only offer a way to train individuals in the life sciences but that also bridge the gap between expert elites and laypeople – the governors and the governed [35–37]. This is a theme that many research participants also picked up on. A 2021 DIY Biology Community Survey participant voices their hopes that DIY biology will lower the entry barriers to science, thereby counteracting misinformation:

“Science access is a pretty big problem, as evidenced by the enormous science misinformation going around. DIY Biology exists because of the lack of science access intersecting with those who want to learn on their own. The barrier to entry is too high for most of the population to get into, which is already a telling problem. But even if they can’t become a PhD, I think accessing the tools and being able to learn the knowledge should be something that is accessible. The Hall of Science and Genspace are two locations I highly credit for satiating my scientific curiosity. If you want to cultivate a society that adores science, is amazed by science, and wants to pursue more of it, then I think those are two very good models to look at.”

It could be argued that hopes of “culturing a society that adores science” glorify science. This touches upon two problems in the identity construction of DIY biology: techno-optimism and the deficit model.

First, DIY biology seemingly carries a techno-optimism that fails to fundamentally challenge the notion that science and technology innovation and entrepreneurship are necessarily of service to society. DIY biologists dedicate themselves to issues that may not get enough academic attention; yet there seems to be a tendency to ignore the social, economic, political, and cultural underpinnings of grand-scale issues such as the climate crisis and antibiotic resistance. These grand challenges will not be solved by technological and scientific progress alone.

Second, DIY biologists occasionally employ the deficit model [38,39], which is based on the premise that laypeople or “amateurs” should acquire a scientific understanding that aligns with that of scientists [39]. The underlying belief is that if the public possessed scientific knowledge, they would be more receptive to novel technologies. However, the deficit model overlooks the fact that science is not rigidly defined, often yields inconclusive results, and is frequently combined with other sources of judgment [38].

#### 3.1.2. Intellectual freedom.

The second sociotechnical imaginary guiding DIY biology is personal freedom and self-development. The movement is characterized by an aversion towards rigid research agendas and the tediousness of only independently running a small fragment of an academic research group’s project. Compared to institutional science, the extra-institutional laboratory seemingly promises a “curiosity-driven approach” [40, p. 4], independence, and agency. DIY biologists imagine that community laboratories will give them a setting in which they can define their goals, play, learn, experiment, observe, direct, and redirect their foci ([41], p. 55).

In her “Biopunk Manifesto”, community leader and visionary Meredith Patterson poignantly articulates DIY biology’s central concerns regarding institutional science and compares an individual’s freedom of inquiry to freedom of speech and religion:

“We reject the popular perception that science is only done in million-dollar university, government, or corporate labs; we assert that the right of freedom of inquiry, to do research and pursue understanding under one’s own direction, is as fundamental a right as that of free speech or freedom of religion” [41].

Meredith Patterson’s statement makes clear how much value DIY biologists place on individual freedom to research and learn. DIY biology centers the individual, allowing them to explore scientific methods and set their personal agendas. Moreover, participants voice that they value DIY biology’s approach to independent and community-led learning and its open-ended, curiosity-driven approach. These principles are imagined to reorder research in a way that favors individuals’ interests and self-development. According to one 2021 DIY Biology Community Survey participant, DIY biology is thought to bring “science back to its roots of exploration” while “answering personal questions and learning more along the way”.

These visions are worth exploring, as they invoke an influential figure: the “modest witness”. The “modest witness” is a concept developed through Shapin and Schaffer’s work [42], which feminist philosopher Donna Haraway formalizes and critiques [43]. Haraway’s work is a reaction to Shapin and Schaffer’s modest witness, who was a wealthy, privileged gentleman. Haraway argues that the scientific observer is not an objective, neutral observer of the world, but rather a situated, partial, and embodied participant in the process of knowledge production [44]. Haraway asks who gets to be modest and whose presence in science can count as modest. In her work, Haraway emphasizes the importance of acknowledging the situatedness and partiality of scientific knowledge, and of recognizing the ways in which scientific observation and experimentation are entangled with cultural, historical, and political factors.

First, community members’ emphasis on individual freedom and the exploration of personal interests in DIY biology indicates an awareness of the situatedness and partiality of scientific knowledge. Rather than approaching scientific enquiry as neutral and objective pursuits, DIY biologists acknowledge the ways in which their own perspectives and interests shape their research questions and scientific methods.

Second, the focus on community-led learning in DIY biology reflects a recognition of the entanglement between social and cultural dimensions and scientific knowledge production. By emphasizing the role of community and collaboration in scientific inquiry, DIY biologists are challenging traditional models of scientific authority and expertise, by emphasizing the role of community and collaboration. Thereby, they may promote a more inclusive and socially aware approach to knowledge production.

Third, the emphasis on curiosity-driven exploration in DIY biology aligns with Haraway’s call for a more humble and self-reflective approach to scientific investigation. By acknowledging the contingencies and limitations of scientific knowledge, and by remaining open to new questions and perspectives, some DIY biologists bear resemblance to Haraway’s “modest witness”, which may help to foster a more democratic and inclusive approach to scientific inquiry. Another survey participant argues that personal freedom and independent exploration does not only benefit the individual but also produces better results:

“I can learn it as I go. And maybe I’ll learn or create an entirely new approach that hasn’t been done in academia before because of their traditional methods and constraints. I can contribute to a project because I’m passionate about it.”

In comparison to the academy, community members commonly imagine DIY biology as an opportunity to work at one’s individual pace, to be creative, to freely choose collaborators and to work interdisciplinarity.

At the heart of DIY biologists’ quest for freedom is a wish to set their own research agendas. This is closely interlinked with the previously mentioned enthusiasm that DIY biology communities have for solving grand-scale problems. One DIY biologist voices that their experience in academia was “only working on a tiny repetitive portion of someone else’s project which I am not allowed to change or investigate new aspects of.” Another individual explains that while working in academia, they felt unsure of who the research they were working on would benefit:

“DIY Biology is free of such constraints as being required to pursue only the flashiest publication worthy experiments and working overtime hours without overtime pay to keep doing something so mundane that might not see any pay-out until a few years and post-docs later. Who knows if this is working? Who knows why it’s not working? Who is this even benefitting? Just the author?”

Moreover, as opposed to academia, DIY biology is thought to make research accessible to individuals independently of the qualifications they hold. Scholarly accounts acknowledge that DIY biologists often assert that every individual should be free to pursue their research interests and set their own agendas, regardless of educational and socio-economic background or institutional affiliations [45,46,47]. They imagine that DIY biology allows for more freedom since membership in a DIY biology community laboratory is easily obtained and independent of academic degrees, prestige, or hierarchies. A survey participant explains that they would rather work in DIY biology than in academia so as to not be “boxed into an extremely limited role due to the lack of a PhD”.

While the strong individualistic tendencies of DIY biology allow for greater personal freedom and self-development, it is not without pitfalls. One of the central problems that are associated with DIY biology is that the imagined freedom comes with a lack of institutional oversight. The identity construction of this extra-institutional science movement hinges on non-conformity; some even claim that the movement is “laden with anti-institution and anti-bureaucracy claims” ([48], p. 171). The DIY biology movement seeks to govern itself from the bottom-up through communal oversight and a loosely shared ethos, which raises immense controversy [5]. Some assert that the community’s effort to mimic “institutional attributes through communal oversight” does not compare to the “professional norms and regulations” that govern “academic and industry labs” [47, p. 427].

#### 3.1.3. Socially relevant research agendas.

In my 2021 DIY Biology Community Survey and the personal interviews I conducted, DIY biologists most often voice that they prefer DIY biology over institutional research because it allows them to solve problems with social relevance. One 2021 DIY Biology Community Survey participant complains that “academia increasingly operates as a business rather than as a knowledge generator”. On the contrary, they imagine DIY biology as “a field that can be self-defining in terms of goals, projects, and collaborations”. DIY biologists often address grand-scale problems, such as the climate crisis, the inaccessibility of scientific infrastructures in the Global South, the unprofitability of treatments for rare genetic defects, and rising prices for pharmaceuticals that make life-saving medications inaccessible.

The communities seek to respond to large-scale, pressing problems, which is why it is perhaps unsurprising that at the start of the COVID-19 pandemic, DIY biologists were quick to set up the OPENCOVID19 initiative. The program attracted more than 4000 individuals seeking to develop “open-source and low-cost tools and methodologies that are safe and easy to use in response to the COVID-19 pandemic” [49]. The hosting organization of this initiative is JOGL (Just One Giant Lab), a non-profit collective of open-source biotechnology enthusiasts seeking to make a change in the world. The makers of JOGL state that their goal “is to catalyze the collective creation of knowledge and solutions to resolve humanity’s most urgent challenges” [49].

Similarly, in personal interviews, DIY biologists often call for scientific agendas that benefit people. More specifically, they aim for research agendas that respond to pressing social and environmental problems and demands. In the minds of many DIY biologists, traditional research institutions are more often aligned with market demands than with social and environmental needs. The DIY biology community’s objective is to solve these overlooked problems. This way, they seek out zones of what has been defined as “undone science” ‒ research that has “potentially broad social benefit” but is “left unfunded, incomplete, or generally ignored” by traditional research institutions [50, p. 2; 51]. For example, the Open Insulin Foundation seeks to innovate a simpler and cheaper way of making insulin [52].

These three envisionings of the DIY biology movement are collectively upheld by its members, reinforced through the movement’s forums and organizations, and publicly enacted by its leaders and participants. As such, they align with Jasanoff & Kim’s [18] definition of sociotechnical imaginaries. The following section delves into how these broadly shared visions are co-produced and adapted within the unique social, political, and historical contexts of three specific countries.

### 3.2. Canada

#### 3.2.1. Regulatory context: DIY biology as a pathway to innovation.

Like many Western nations, Canada began promoting biotechnology as a key area for economic growth in the 1980s. The introduction of the 1983 National Biotechnology Strategy prioritized innovation and industrial leadership, with regulation as a secondary concern [53]. Consistent with this orientation, Canada follows what Jasanoff terms a “products” approach; the country governs biotechnologies with the assumption that risks are not unique in a way that would require biotechnological products to be “treated any differently from similar products created by traditional biological or chemical processes” [54, p. 315]. A central principle of Canada’s biotechnology regulatory framework is that “existing legislation and regulatory bodies” govern biotechnology products by “build[ing] on existing laws and expertise, rather than developing entirely new laws and agencies” [55, p. 1].

The historical framing of biotechnology affects its approach to DIY biology in two main ways. First, regulators adhere to a “burden of proof” approach, which implies that DIY biology is safe until proven otherwise (Canadian Regulator, Interviewee #3). Second, the tradition of emphasizing innovation over risk continues in how DIY biology is imagined: compared to Germany and Great Britain, the movement is envisioned more positively, as a pathway to entrepreneurship and innovation.

In Canada, the socio-political environment can be described as broadly supportive of DIY biology. In a regulatory context, it is framed within the context of a national innovation strategy [56, p. 4]. The high cost of post-secondary education and an emphasis on entrepreneurialism have fostered a cultural narrative of the “garage innovator”, who is thought to be an innovator working outside institutional boundaries to develop technological solutions. Reflecting this, the federal government has maintained a permissive regulatory stance and actively positioned DIY biology within its national innovation strategy [56] As the Public Health Agency of Canada notes, policymakers have not only refrained from restrictive oversight but also provided support in the form of expertise-sharing and networking infrastructure [56]. DIY biology is imagined as a means to democratize science, boost STEM learning, and generate novel responses to societal challenges like food security [55,57]. These expectations position DIY biologists less as hobbyists than as grassroots contributors to the bioeconomy, which highlights a vision where bottom-up experimentation complements national scientific goals.

#### 3.2.2. Democratization biology by creating alternative epistemic spaces.

My research shows that Canadian DIY biologists commonly frame their work as a form of democratizing biology by making research infrastructures more accessible, challenging expert-public divides, and promoting open-source ownership models. These visions are rooted in a belief that broader participation in science will accelerate innovation. Much like regulators, DIY biologists imagine themselves as contributors to the national bioeconomy and drivers of inclusive, grassroots innovation.

**Accessibility of research infrastructures: Back to former times:** DIY biologists do not aim to change the methods of science as much as the sites at which it is conducted. This is grounded in visions of correcting grievances associated with traditional research institutions. Accordingly, Canadian community members would sometimes explain that this aligns alternative epistemic spaces with science in former times, thus freeing scientific methods from research institutions:

“If you think about the history of science, you know, particularly like in the United Kingdom, you know, gentleman science. […] A lot of the patrons of science […] had their labs at their homes. So, institutions came later, and science wasn’t done in institutions in the beginning. And people have to separate the concept of the scientific method from the site that science is done.” (Canadian Community Member, Interviewee #18)

Like this interviewee, Canadian DIY biologists in general are very vocal about their vision of creating more accessible, and thus “better”, epistemic spaces. In doing so, they favorably mention gentlemen science, which is somewhat paradoxical, as I explain in the concluding section.

However, this statement indicates that community members view academia as simply not accessible enough. They argue that extra-institutional science spaces enable them to focus on research without the extra hurdles of having to access traditional funding routes or being pressured to publish research papers. One community member states that this makes DIY biology “more part of the world”:

“Some of these people are just like, really, really, really good at what they’re doing. And they’re doing it in their garage. And they might not have funding for certain things […], they might do it on their own dime. Or maybe they have a hard time publishing because of their background or whatever. Yeah, so I think the DIY approach kind of like disseminates it a little bit better into the world, because it’s more a part of the world than it is in academia.” (Canadian Community Member, Interviewee #23)

As the statement above indicates, these imaginations of DIY biology as a better, more accessible, epistemic space are closely tied to academia-critical sentiments. While academia is envisioned to “gatekeep” and slow down scientific exploration, DIY biology is thought to democratize biology by making it accessible to everyone, independent of their background. This interviewee explains how they imagine DIY biology’s hands-on approach to better convey biological futures:

“And all of the gatekeeping that is associated with planning a bio project […] becomes slowed down quite a bit through academic processes. […] I think it’s important to be able to open it up to everybody and bring it to people where they can try to understand how it will affect the future through their own understanding of it […] and they can understand it better with their own hands-on experience.” (Canadian Community Member, Interviewee #23)

Moreover, the findings indicate that Canadian DIY biologists share a strong belief that making research infrastructures accessible will advance scientific progress. The underlying assumption here is that traditional scientific institutions are not a necessity; scientific advancement might as well be made in alternative epistemic spaces. This interviewee explains that if the potential of community biology is not harnessed, then societies might miss out on at large.

“There have been community […] efforts that have led to great advances, like, wasn’t it two scientists in Toronto who […] made insulin useful? That was […] in a very small lab, you know. […] That was a huge discovery that I feel, you know, someone… a scientist working independently is able to do. And a lot of science doesn’t do that anymore. And so, so there’s stuff that we could be missing out on understanding, right. Like, I feel like that understanding that science isn’t necessarily confined to institutions, and that a lot of scientists are able to achieve things if they’re just given the resources. And, and, like limiting those resources is a net negative for everyone.” (Canadian Community Member, Interviewee #24)

This DIY biologist refers to a small team of scientists at the University of Toronto who made decisive advances in the development of insulin in the early twentieth century [58]. The interviewee highlights the independence that these scientists were thought to have. In fact, visions of individuals, including so-called garage entrepreneurs, seem especially prevalent in the Canadian context. It seems that DIY biologists’ approaches to democratization include imaginaries as much as practices.

A closely related vision is that of DIY biology challenging the expert-publics divide, which is the topic of the following investigation into Canadian DIY biology.

**Expert-publics divide: Envisioning cheaper education:** Like their German and British counterparts, Canadian DIY biologists envision their movement as one that is democratizing education, thereby challenging the expert-publics divide. Canadian DIY biologists particularly express that they perceive DIY biology as an avenue to learn within one’s own budget. It should be noted, however, that this would not necessarily facilitate democratization. When individuals draw on their own financial means to get an education, science remains a relatively privileged economic undertaking.

Several Canadian community biologists shared that they felt motivated to join a community laboratory to gain lab experience after they had already completed an undergraduate degree in the natural sciences. One contributing aspect might be Canada’s high price tag of university-level education, at least compared to Germany [59]. However, in Great Britain, higher education is equally, if not more expensive (ibid.), but visions of DIY biology as a cheaper alternative to higher education are not nearly as prevalent as in Canada.

One Canadian interviewee explains that they prefer community laboratories as a learning environment because getting a graduate degree would put too much financial strain on them. Instead, they found the community laboratory to be an effective way to learn in their own free time and at their own pace:

“I think that, like, that’s the most important thing for me to this is that it allows people who to grow at their own pace, and you know, what, their own budget. And I think that that’s really cool.” (Canadian Community Member, Interviewee #24)

Moreover, DIY biology is envisioned to emulate (what are thought to be) scientific learning environments of the past. Even though they have not (yet) pursued graduate education, the same community member explains that they value DIY biology for its “system of apprenticeship” as opposed to contemporary academia, which is thought to be too constrained:

“I think that that’s something that’s really missing in […] you know, science. It used to be more of […] a system of apprenticeships and the like, right. And now it feels so… it doesn’t feel like that anymore. And I think that that’s what’s really cool about open science and biology is that it gives people access who otherwise for one reason or another, just haven’t been able to work within that very constrained system.” (Canadian Community Member, Interviewee #24)

One community organizer claims that university laboratories are even inaccessible to the university’s members themselves. They cite liability issues and a lack of laboratory training throughout undergraduate degrees as two reasons for why they think universities deny students laboratory access:

“University students are screaming to get access to space in the universities, but the universities don’t want to give it because of liability issues. They don’t trust the undergraduates. […] When they go into graduate studies, they come under a principal investigator with his [sic] team, and that’s how they get training. Whereas underneath that, universities don’t offer that type of structure. And so, community labs are a great resource.” (Canadian Community Member, Interviewee #18)

Many community members share the vision that DIY biology is more effective at public engagement than universities are. This interviewee shares this opinion, using the COVID-19 pandemic and vaccines as an example to illustrate their point:

“So, I think there’s a huge opportunity […] to disseminate some of the molecular biology knowledge in the regular population. Like, if you’re a truck driver, who doesn’t want to do a vaccine, because you heard it from the lady […] from the end of the village, then it is very unlikely that you’re going to pay, you know, $30,000 a year to go study molecular biology to understand […] viruses. But there is a chance to, you know, maybe drop into a session [at a community laboratory] where somebody talks about viruses or shows you something about viruses. [Covid showed that] better education in the population would help society in general. So that’s one of the things I think that should be some done because universities cannot reach... or at least it cannot be proven that they are very efficient at reaching non-students.” (Canadian Community Member, Interviewee #21).

DIY biology community laboratories tend to be populated by individuals with an academic background in the natural sciences. Thus, it is doubtful how much success DIY biologists are having in reaching out to publics, such as the above-mentioned truck drivers.

Moreover, in the context of DIY biology’s education capacities, some Canadian community members would sometimes argue that they dislike public reluctance towards specific biotechnologies. They most often brought up genetically modified organisms (GMOs) and skepticism towards the COVID-19 vaccines as examples. Their underlying assumption is that if publics knew more about biotechnology ‒ and science and technology more generally ‒ they would be more accepting towards (novel) technologies. Community members claim that the interaction between them, as trained scientists, would enable laypeople to learn how to understand science “correctly”, i.e., like scientists [39].

**Open-source biology: Collaborative production:** Moreover, DIY biologists aim to democratize biology by advancing open-source ownership regimes. Open-sources resources are imagined that place collaborative production and learning over private ownership. One interviewee explains how their work on an open-source biology collection would not lend itself to entrepreneurial pursuits but might democratize who can access scientific resources:

“If I do anything in his lab, I can’t take that and then immediately turn it into a business because that’s for everybody, right? And so, what I’m working on now […] this is going to likely be part of [an open-source biology] collection, for all scientists to access and all that… all the information is for everyone, but none of it proprietary.” (Canadian Community Member, Interviewee #24)

Similarly, DIY biologists compare the computer sciences and (synthetic) biology. One interviewee points out how they imagine the parallels between biology and the development of open-source hardware:

“I am a big believer in open science, and I look at the history of open-source software. How […] if we didn’t have open-source software that has transformed everything, how would we do anything? Where would the world be? We would be locked in all these proprietary systems, you know, […] Microsoft web servers, where in order to put up your website, you’d be paying thousands of dollars […] And I see the potential for that transformation, but not to the same extent, with biology… with synthetic biology. So, you know, it’s one of those balls that I’m pushing up the hill. And hopefully, it will lead to something.” (Canadian Community Member, Interviewee #18)

This community member express that they are not sure if synthetic biology will fundamentally transform the field of biology. Nevertheless, they seem eager to pursue DIY biology to advance an open-source approach to synthetic biology, meaning that they were also working on an open-source collection of biological parts.

There is evidence that DIY biologists are in fact making strides in the development of open-source biology. For instance, an open-source universal connector method of genetically engineering yeast was developed at a community laboratory in Vancouver [28]. The Open Yeast Collection is thought of as “a foundational and enabling framework for contributing to the creation of an open, sustainable and equitable bioeconomy” [60]. In line with the other two themes, open-source ownership is thought to democratize biology by facilitating the enrolment of additional participants. Accordingly, the Open Biofoundry is advertised to “appeal to a wide variety of users from educators & students, community-based & academic researchers and bio-entrepreneurs [60].

Moreover, DIY biology is not only thought to democratize scientific research, thereby enrolling additional participants, it is also thought to offer greater intellectual freedom to its participants than traditional scientific institutions, as I detail in the following section.

#### 3.2.3. Intellectual freedom: Prioritizing self-determination.

The second key imaginary of DIY biology is personal freedom and self-development. The movement at-large is characterized by an aversion towards rigid research agendas and the tediousness of only independently running a small fragment of an academic research group’s larger project. Compared to institutional science, the extra-institutional laboratory seemingly promises a “curiosity-driven approach” [40, p. 4], independence and agency.

Out of all three countries under investigation, this imaginary is most often invoked in the Canadian context. Like this interviewee, Canadian community members often voice that they value DIY biology for prioritizing self-determination:

“That self-determination aspect of DIY bio, to me, it’s quite central. […] I don’t want to depend on an institutional set of priorities to determine my access to medical biology. I also don’t want to depend on a capitalist, industry driven set of priorities determine what genetic modifications we make in the material around us.” (Canadian Community Member, Interviewee #23)

Canadian DIY biologists, like the individual cited above, want to take matters into their own hands. This is to say that they want to set their own research agendas, independent of the priorities and funding regimes in industry and academia.

This, of course, implies a critique of traditional research institutions. Looking back at their career in academia and industry, a DIY biologist states that they prefer the movement over industry, since it allows a curiosity-driven approach. They started out in academia, then moved to industry and eventually founded a community biology lab:

“I am more suited to curiosity-driven science rather than just cranking nuts and bolts. […] And so, I ended up leaving the company. […] And I’ve always had this passion for biology. And so that’s why I decided to start my own lab.” (Canadian Community Member, Interviewee #18)

Community members express that DIY biology gives them the freedom to pursue everything that they want, from entrepreneurship to hobbyist level exploration:

“I kind of see community biology [...] as an alternative place to be able to do whatever it is that you want to do. And that could be being an entrepreneur, where you can actually come in, you have access to resources that you wouldn’t otherwise have, or that you need a shitload of money from the investors to be able to get. And you can test ideas. I remember I was down at [a synthetic biology] conference way back in 2015. And then [the speaker] used the phrase, “credit card and a dream”. And I thought, wow, that’s totally appropriate.” (Canadian Community Member, Interviewee #18)

Some Canadian DIY biologists seem convinced that intellectual freedom is key to innovating in their field. This ties into Canadian DIY biologists’ vision of entrepreneurship, specifically a “credit card and a dream” narrative, which conveys that one only needs courage and little resources to pursue a dream, and eventually see success. As noted above, this would include a very particular, privileged demographic that could then undertake DIY biology.

In other respects, Canadian DIY biologists’ visions of their movement also resemble that of the movement at-large. This is to say that the following investigates how Canadian DIY biologists, like their counterparts in Great Britain and Germany, imagine their movement to foster socially relevant research agendas.

#### 3.2.4. Socially relevant research agendas: Getting important things done.

Like DIY biologists in other countries, Canadian interviewees mention how they envision DIY biology to address socially relevant research agendas. One Canadian community organizer explains to me that “DIY biology has amazing potential to attract people’s attention and to get some extremely important things done.” (Canadian Community Member, Interviewee #21). This is to say that Canadian DIY biologists would sometimes point out that their movement can address “undone science” [50].

This imaginary of DIY biology’s socially relevant research agendas is often related to a critique of traditional scientific research institutions. When I asked DIY biologists if they thought that DIY biologists can accomplish something that institution-based researchers cannot, I would at times get passionate replies. This was especially true for Canadian interviewees. DIY biologists often argue that universities are too bureaucratic, while industry corporations are monetarily motivated, thus failing to address pressing problems. One interviewee makes this point using the development of new drugs as an example:

“In universities, for instance, how many drugs did the university […] create in the past that you know of, in your life? Probably zero, right? So, what’s happening there is… if you go into the companies, for instance, let’s take the healthcare sector, because that’s an easy one to talk about […]. If you take a company and let’s, say that somebody has a product that will save peoples’ lives, the decision is made, first of all, the decision is made solidly on financial criteria. If that drug will not bring money to the company […] the company will not start doing that.” (Canadian Community Member, Interviewee #21)

The interviewee holds that they think that traditional research institutions do not sufficiently address problems of social relevance. They maintain that companies are primarily financially motivated. Hence, they are of the opinion that DIY biologists are better suited to address research areas of social relevance, such as the development of new drugs.

However, I also want to note that not all interviewees are convinced that DIY biology is better suited to address socially relevant research problems. Some assert that DIY biology is simply too limited in its resources as compared to traditional scientific institutions. When one interviewee mentioned the Open Insulin Initiative, I asked them if they think DIY biology is better at addressing societal problems than institutional science. They responded, “Oh, hell no. I think, though, that DIY bio puts more control in the individuals’ hands” (Canadian Community Member, Interviewee #22). This statement again points towards another key imaginary, which is that DIY biology, and its alternative epistemic spaces, allow for greater intellectual freedom than academia or industry research laboratories.

While Canadian DIY biologists hold strong and hopeful imaginaries, the next chapter examines how these visions meet the practical realities of regulatory frameworks, institutional dynamics, and resource limitations.

#### 3.2.5. Making Canadian DIY bio: Between freedom and compliance.

Canadian DIY biologists often face practical challenges that complicate how their visions are put into practice. DIY biology laboratories may operate outside traditional research institutions but must still meet the same regulatory requirements as academic or industry labs. Establishing spaces that comply with these regulations is often costly, especially in large cities where many communities are based and where real estate and overhead costs are high. This mismatch between DIY biologists’ desire for independence and the demands of formal compliance highlights one of the many tensions they must negotiate.

Canadian DIY biologists frequently report financial limitations as their primary challenge. Many rely on personal savings or informal funding, embodying the ethos of the “credit card and a dream” garage entrepreneur—a recurring figure in Canadian narratives about innovation. However, this model limits who can meaningfully participate. Despite claims of openness, one must have discretionary income to join or self-fund, meaning participation is not equally accessible. Access to suitable lab equipment and reagents is uneven; while some communities improvise with repurposed materials or open-source tools, financial constraints remain a persistent barrier.

Although DIY biology promotes public engagement, many community labs primarily attract individuals with university-level training in the natural sciences. This raises questions about the extent to which DIY biology truly democratizes science. While it aims to lower barriers, in practice, it often reproduces existing exclusions—financial, educational, and social. The movement’s nostalgic appeal to the “gentleman scientist” may inspire, but it also risks reinforcing privilege by assuming access to time, resources, and prior knowledge.

Developing open-source biotechnologies is difficult, and even when successful, questions remain about whether, and how, they should be monetized. One example for Canadian DIY biology-inspired entrepreneurship is the Saskatchewan-based firm Amino Labs, which markets DIY biology education kits [61]. The company aims to solve a common problem among DIY biology communities, which is a lack of trained mentors to onboard and train newcomers. Amino Labs seems to incorporate DIY biology’s ideal to democratize biology by making spaces and educational resources more accessible.

While intellectual freedom, democratization, and socially relevant research remain central visions, DIY biology communities are deeply shaped by material constraints. Regulatory compliance, funding challenges, and internal governance shape what kind of work is possible and who can do it. The tension between DIY biology’s ideals and its practical realities reflects an ongoing process of negotiation, trade-off, and adaptation within the Canadian context.

The Canadian case offers insight into the constraints and contradictions facing DIY biology efforts. A closer look at Great Britain reveals how these tensions manifest differently in a context marked by informal oversight, high compliance barriers, and limited state engagement.

### 3.3. Great britain

#### 3.3.1. Regulatory context: An informal approach to DIY biology.

Great Britain has historically taken a broader, precautionary approach to biotechnology regulation compared to Canada, with early risk evaluation involving expert advisory bodies such as the Genetic Manipulation Advisory Group (GMAG) and the Department of the Environment [54,62]. This evolved into more formalized processes under the Environmental Protection Act (1990) and the establishment of the Advisory Committee on Releases to the Environment (ACRE), which centralized GMO reviews while incorporating more diverse perspectives, including an environmental representative, to balance industry growth with safety concerns [63,64].

Unlike the attention seen for biotechnology broadly, DIY biology in Great Britain has been approached more informally and with less public deliberation. Regulation is primarily overseen by the Health and Safety Executive (HSE), which maintains sporadic, informal contact with DIY biology communities and gathers covert “intelligence” to monitor potential biosecurity risks (British Regulator, Interviewee #2). This cautious but low-profile regulatory stance contrasts with more explicit and public scrutiny in other countries, such as Germany. The effort to collect intelligence indicates that DIY biology may be framed as a potential biosecurity risk.

Policy stakeholders such as the Nuffield Council on Bioethics, the Parliamentary Office of Science and Technology (POST), and the Royal Society of Biology contribute to evaluation and decision-making in this context. Although all of these entities have published some policy-relevant materials on the movement, compared to the other countries under investigation, DIY biology is (thus far) not a major focus of formal policymaking or public debate. This reflects a regulatory culture that prefers expert-led, case-by-case assessment over broad public engagement. However, to understand how these regulatory dynamics are experienced and negotiated by practitioners themselves, it is crucial to turn to the imaginaries that guide their work.

#### 3.3.2. Democratization: Closing the gap.

The by far most prevalent imaginary among British DIY biologists’ centers around the democratization of biology, which include a call for accessibility of research infrastructures, a challenge to the expert-publics divide and the formation of open-source ownership regimes. The democratization of biology is thought to aid the enrolment of a wider range of individuals and groups, thus spurring innovation.

I find that British DIY biologists mostly envision democratization following two themes: First, DIY biology is envisioned as a space that allows individuals to access biological and biotechnological research. Second, the movement is imagined as making biotechnological and biological education more equitable by granting a wider audience access to courses, hands-on learning, and resources. The analysis indicates that open-source ownership regimes are more of a sidenote in British imaginings of the DIY Biology movement, which is why this theme or vision is not included in this analysis. However, it should be noted that British DIY biologists do partake in open-source research and projects. In personal interviews the topic of open source was just not mentioned with nearly the same frequency and emphasis as in the two other countries under investigation.

**Accessibility: Reducing start-up costs:** British interviewees often made it clear that they deem the institutional pathway of realizing a project in industry or academia to be too bureaucratic, expensive and time consuming. DIY biology, as an extra-institutional science movement, is envisioned as a more accessible epistemic space. Like their Canadian counterparts, British DIY biologists are often critical of established scientific institutions. They tend to emphasize that getting access to a university or industry lab is not only difficult but also is thought to force a researcher to align with the institutions, e.g., a university’s mission:

“I think if you’re someone who has a project in mind, I mean, the main advantage of DIY bio is the accessibility of the places, because it’s very difficult […] to get access to university labs without having a direct collaboration with the university. I mean, collaboration means funding, which means a lot of justification as to the work that you’re doing and how it’s kind of linked to the mission of the university. Or if you want to get into industry labs there’s a massive gap […] it’s very expensive and that’s problematic. If you’re not doing this as a as a venture with investment […] there is basically nowhere that you can do that.” (GB Community Member, Interviewee #11)

In fact, many British DIY biologists deem biology to be so impactful that everyone ought to have access to its resources. One interviewee argues that biological experimentation should be as easily accessible to citizens as fixing up a bicycle.

“As citizens, they can fix bikes […] up in their shed. If you want to, you should be able to have access to actually building things with biology, because biology is really powerful, right.” (GB Community Member, Interviewee #10)

Other British DIY biologists share the notion that biology is a powerful tool: They often stress the social benefit of making biology accessible to as many individuals as possible, especially to those without an institutional affiliation.

Compared to the computer sciences and information technology, biotechnology is imagined as less accessible due to the high start-up costs. This is where DIY biology is imagined as closing a gap, even for those who are employed at institutional research laboratories:

“Even if you in your day job have access to a lab, you’re not going to be able to use it for some random hobby project or for a new venture that you’re starting up because it’s a lab that’s built for a purpose and you’re probably employed to work in it for a purpose. And so, there’s kind of very little flexibility compared to […] the IT space and you can just work on it on your evenings and weekends, and you don’t really need to invest a lot of money or even in hardware. […] And so, the biology there is very, very much a gap.” (GB Community Member, Interviewee #11)

In my research, I almost exclusively encountered British DIY biologists who hold qualifications in the natural sciences and most often are affiliated with traditional scientific institutions in industry or academia. This, again, shows that DIY biology is an extra-institutional science movement in which professionalized communities and initiatives seek to create scientific knowledge outside of traditional academic, industry or military research institutions. It is particularly interesting that although they have access to research institutions, they still feel constrained and thus seek out alternative epistemic spaces.

In fact, British DIY biologists were especially vocal about the gap that their alternative epistemic spaces are thought to close. Over the course of this research project, I encountered many British DIY biologists who explain emphatically that they would not be able to pursue their projects if it wasn’t for accessible DIY biology communities, their spaces, resources, and support. They explain that the community biology laboratory provides the key element, as renting an industrial research laboratory for hours at a time would be too costly. Moreover, many contended that the necessary equipment would be too expensive to buy, even second-hand. They also explain that the community laboratory made materials and reagents available that they would normally not be able to order to their home address, due to biosafety and biosecurity regulation. The individuals I talked to enthusiastically explain that they thought that their membership fees were very affordable for the quality tools and resources at their respective community laboratories.

**Expert-publics divide and sharing a passion for science:** The second most prevalent vision among British DIY biologists is that DIY biology democratizes the sciences, thereby challenging the experts-publics divide by providing an accessible avenue to education. British DIY biologists share the vision that biology, and the natural sciences education more generally, is easy to learn and should be accessible to anyone, independent of their personal circumstances and academic background. One interviewee passionately explains that an interest in zoology and botany first sparked their interest in studying biology; a passion that they believe many others share.

“It’s like, I want to understand how to grow these things, how biology works […] about sustainability in an ecosystem and species. And I think that a lot of people want to learn more.” (GB Community Member, Interviewee #10)

British DIY biologists, most with relevant degrees in the natural sciences, seem to share the belief that those without institutional qualifications and affiliations want – and should – be able to learn about biology.

British DIY biologists, like their Canadian and German colleagues, tend to compare biology to other fields that that they deem more accessible. Most often, community members compare biology to fields and skills such as information technology and making/handicrafts. In doing so, British interviewees often emphatically explain that it is easy to grasp the basic tenets of biology and that everyone can successfully learn and apply popular wet lab techniques. DIY biology is envisioned to enable individuals to participate in biological research and experimentation without going through any institutional degree and training programs:

“So, we’re focusing more on the DNA analysis, but there are some things that are not technically that difficult. They can be learned, and they can be hugely valuable.” (GB Community Member, Interviewee #16)

They imagine that DIY biology-inspired tools and resources can give individuals the necessary tools to participate in biological research. One participant explains this using the example of a popular laboratory technique, the PCR [polymerase chain reaction].

“What we’re really trying to do is […] give someone confidence that they can do it by themselves and take away this notion that this PCR is something that only someone who’s gone to university for ten years, as has done several postdocs, could do, because this is not I mean, I’ve seen many people do that, learn this [PCR] in an afternoon and produce great results.” (GB Community Member, Interviewee #16)

They deem that wet lab skills, such as PCR, can be easily learned and do not require institutional qualifications. In fact, community laboratories often offer workshops that aim to teach beginners wet lab skills and techniques. British interviewees were especially vocal about their confidence that biology is not too difficult to learn for members of the public.

As I mentioned, DIY biologists tend to envision that democratizing biology will help to enroll additional participations, which is then thought to spur innovation. In conversation with British DIY biologists, it is not initially clear what exactly they imagine other, additional benefits of democratizing biology to be. However, when quizzed, research participants argued around two central themes: First, most often, they contend that by democratizing biology, and thereby making biology education more accessible to individuals, they become more knowledgeable, curious, and thus empowered (and potentially innovative). Second, community members argue that making citizens understand DIY biology better would help them to become more accepting of certain technologies, such as genetic modification.

#### 3.3.3. Intellectual freedom: Science beyond the priesthood.

The vision of DIY biology aiding democratization is related to a second imaginary: that DIY biology fosters intellectual freedom. This is the least dominant imaginary among British DIY biologists. When DIY biologists invoke this imaginary, they imagine intellectual freedom along two lines: Freedom from institutional bureaucracy and academic/industrial research agenda and freedom from the interests and ties that come with traditional funding avenues (i.e., academic grants or corporate sponsorships). This notion also becomes apparent in the statements on the previous pages: DIY biology is thought to give a greater number of individuals the freedom to choose the trajectory of their project freely, independent of an institution’s research agenda and the need to secure financial resources first.

To be specific, institutions are sometimes thought of as a limiting factor to an individual’s intellectual freedom. Critical of traditional institutions, one interviewee compares academics and industry-based researchers to the “priesthood”:

“There’s no reason why that should be restricted to only a specific kind of priesthood, you know, academics or industry people. So, I think it’s that belief that that there is a social benefit in making some of these things much more cheap and much more accessible.” (GB Community Member, Interviewee #16)

Again, making biology more accessible is thought to foster more freedom, which is thought to be socially beneficial.

Perhaps British DIY biologists do not invoke the imaginary of DIY biology fostering individual freedom very often because they are more focused on collective rather than individual benefits. This is to say that the visions of British DIY biologists in relation to their movement were typically not centered on an individual’s freedoms, for example their personal freedom and self-development, but were rather focused on the larger collective’s benefit. They also hardly ever spoke about the ways in which they as individuals benefit from DIY biology, but rather imagined DIY biology as a collective endeavor. Examples include publics who were imagined gaining access to laboratories and researchers in the Global South who would gain steadier supply chains and cheaper, more easily accessible equipment.

Compared to their Canadian counterparts, British DIY biologists less frequently imagined DIY biology as an avenue to gain intellectual freedom in order to subsequently launch commercial ventures. Similarly, out of all three countries under investigation, British DIY biologists hardly ever envisioned their movement as a means to advancing innovation in biology and/or biotechnology. Instead, they envisioned their activities as community-focused projects driven and characterized by curiosity, experimentation, and play. Some DIY biologists even framed commercial ventures within community laboratory settings as something that is “other”, i.e., not part of DIY biology. Commercial ventures were sometimes even thought of as a necessary, yet undesirable, way of keeping a community laboratory afloat financially.

#### 3.3.4. Socially relevant research agendas and US DIY biology as other.

I describe a third imaginary at the core of the DIY biology movement, which also features in British DIY biologists’ envisioning of their movement: socially relevant research agendas.

In the 2021 DIY Biology Community Survey I conducted DIY biologists most often voice that they prefer DIY biology over institutional research because it allows them to solve problems with social relevance. In interviews with British DIY biologists, they invoked the imaginary of a socially relevant research agenda less often than the imaginary described above (i.e., the democratization of biology).

Interestingly, several British DIY biologists brought up the imaginary of DIY biology’s socially relevant research agendas specifically in a US context. While some British DIY biologists deem the US regulatory system to be beneficial to the democratization of biology, the US DIY biology-sphere’s otherness was also envisioned with a cautionary tone. This is to say that participants imagined the work of US DIY biologists to alleviate political and social grievances. Interviewees frequently mentioned the Open Insulin Project specifically and the inaccessible and highly neoliberalized US health care system more generally [52]. Interestingly, the oft-shared imaginary of DIY biology as a movement that alleviates social, political grievances, e.g., in medicine, is specifically invoked in the US but not in a British context. Again, much of the focus of British DIY biologists seems to lie on democratizing biology by making its spaces, resources, and tools more accessible.

This is not to say that socially relevant research agendas do not matter to British DIY biologists. They sit at the very core of the movement’s identity and matter greatly to community members in Great Britain. However, when British DIY biologists spoke about the potentials and advantages of their movement, this imaginary received less attention than the democratization of biology.

In the context of socially relevant research agendas, British DIY biologists again focused on their vision of making biology research more accessible. In fact, there are several groups and individuals in the country working on tools that would make biotechnological research more accessible for researchers in other countries, specifically the Global South. Several initiatives were first initiated at Cambridge University and focus on hardware development and addressing reagent supply issues that often make research in a Global South context more difficult [65].

#### 3.3.5. Making british DIY bio: Navigating collective benefit.

One of the most commonly reported challenges by British DIY biologists is the difficulty of building and sustaining active communities. Many community organizers describe being overextended, facing persistent financial constraints, and struggling to provide adequate training opportunities for new members. While British DIY biologists strongly articulate their belief in the democratization of biology, emphasizing that biology is not too complex to learn, they also acknowledge the significant demands involved in onboarding and training newcomers. First, there is a shortage of qualified volunteers able to offer consistent guidance. Second, the level of individualized attention required to bring new participants up to speed is high. As a result, the community’s vision of an inclusive, accessible science often clashes with the reality of limited resources and volunteer burnout.

This contradiction underscores a broader tension: while biology is envisioned as open and graspable for newcomers, in practice, meaningful participation often requires substantial technical training and mentorship, resources that are unevenly available in the DIY sphere.

British authorities, namely the Health and Safety Executive (HSE), have adopted a largely hands-off, observational approach. Instead of sustained engagement with DIY biologists, regulators tend to rely on academic intermediaries or occasional, informal contact to stay informed about developments within DIY biology communities. This passive stance contrasts not only with the more proactive support seen in Canada but also with the ambitions of British DIY biologists themselves, who envision their work as a driver of innovation and education.

British DIY biologists often frame their work as an opportunity to conduct research outside the constraints of institutional science. However, this vision is somewhat limited by the UK’s strict regulatory requirements for laboratories. Community members report that it is labor and cost-intensive to adhere to regulation, which challenges their aspirations for accessible, unbureaucratic spaces for learning and experimentation.

Community members noted that the entry barriers are very high in Britain particularly for those hoping to explore genetic engineering. Post-Brexit, some DIY biologists hoped for a more permissive regulatory environments akin to those in North America. While consultations between the HSE and community labs have occurred, officials reiterated that no changes to key legislation, such as the Genetically Modified Organisms (Contained Use) Regulations 2014, are planned. As such, DIY biologists continue to operate within a tightly bounded legal framework, particularly when working with genetically modified organisms (GMOs), which require specialized, registered lab spaces that British DIY biology communities do not have the resources to set up and run.

Although DIY biology is occasionally acknowledged by government bodies, such as HSE visits to community labs, this recognition tends to be sporadic rather than substantive. DIY biologists may be consulted from time to time, but there is little evidence of structural support or meaningful integration into broader biotechnology governance or even industry. This dynamic leaves DIY biologists in a position where the community members and their laboratories are visible to the state, but not quite empowered by it. While their presence is acknowledged, their aspirations for more open, accessible science remain largely unrealized within current regulatory conditions.

Having analyzed how DIY biology is imagined and materialized in Canada and Great Britain, I now want to turn to the third country of investigation: Germany.

### 3.4. Germany

#### 3.4.1. Regulatory context: The precautionary principle.

In contrast to the more market-driven North American burden of proof approach, biotechnology regulation in Germany is strongly shaped by the precautionary principle. This principle holds that in situations of uncertainty, potential risks should be treated as if they were factual [66]. In keeping with this approach, German regulators typically avoid making potentially controversial decisions without legal backing and thorough risk evaluation [67, p. 279; 68]. Early governance frameworks, such as the Genetic Engineering Act (Gentechnikgesetz) of 1990, reflect this stance by introducing strict oversight of genetic modification activities [67].

Germany thus has a long-standing tradition of rigorous biotechnology regulation and extensive bureaucratic assessment procedures [54,69]. Compared to Canada, there is a more visible “resistance to experimentation with new forms of life, in nature, society, or the state” [67, p. 275]. This tendency is mirrored in Germany’s policy discourse on DIY biology, which is predominantly focused on the discussion of potential risks and dangers.

The regulatory treatment of DIY biology exemplifies how established legal and bureaucratic processes are triggered in response to emerging developments in science and technology. Official scrutiny of DIY biology began in 2009, when institutions such as the German Research Foundation (DFG), the German Academy of Science and Engineering (acatech), and the National Academy of Science (Leopoldina) began framing it within broader debates about synthetic biology [70]. Their joint report evaluated both the risks and promises of synthetic biology, setting a cautious and critical tone that has shaped Germany’s approach to both fields ever since.

This regulatory culture directly influences how DIY biology is practiced and perceived. Most community laboratories operate at the lowest biosafety levels and avoid genetic modification altogether. Despite these restrictions, DIY biology in Germany continues to thrive in grassroots settings that creatively work within legal constraints while promoting accessibility, collaboration, and social impact. Its emphasis on sustainability, intellectual autonomy, and public participation reflects both a response to institutional limitations and an alignment with Germany’s broader socio-political values of precaution and responsible innovation.

Building on this country-specific backdrop, the following section evaluates how German DIY biologists imagine their roles and purposes.

#### 3.4.2. Democratization: Experimenting beyond the university.

Like their counterparts in Canada and Great Britain, German DIY biologists imagine their movement to democratize biology. More specifically, they imagine that DIY biology can make research infrastructures more accessible, challenge the expert-publics divide and aid in the formation of open-source ownership regimes.

**Accessibility: Making room for experimentation:** Like their counterparts in Canada and Great Britain, German DIY biologists often argue that traditional institutional research spaces are not accessible enough. Moreover, like community members in Great Britain and Canada, they are most often educated in the natural sciences and/or are affiliated with a university, e.g., as a student or researcher.

Nevertheless, they, too, deem it necessary to build alternative epistemic spaces outside of traditional research institutions. DIY biology spaces are thought of as filling this gap by providing alternative, more accessible spaces.

One community organizer, who has observed the steady influx of university-affiliated individuals to DIY biology, speculated about possible motivations to join the movement:

“The community has grown tremendously […] people come from academia and all different kinds of fields. They join because they find our [community biology] topics exciting. They are keen on having room for experimentation.”

The same organizer also quite passionately explained how they imagine community biology spaces to function:

“It [DIY biology] is not a niche phenomenon, it really is a room for experimentation and a room for skills that we will need in the future. There is a huge need, in our generation, to not just talk but to address things hands-on. It’s political […] it is about empowerment. This [the DIY biology community] is a hotbed for different ideas, innovation, and a place for education […] it offers something you just do not find at university, right?” (German Community Member, Interviewee #28)

Their statement invokes a number of different prevalent visions: that DIY biology, by making alternative epistemic spaces, helps to address pressing problems and offers educational opportunities. Much like British and Canadian community organizers, this interviewee deems this a combination not currently offered by universities:

“I think that there are so many people at universities that are interested in participating in genuine science competitions. But it is just not done that often. It is not part of the university curriculum, which is already stuffed. Projects cost additional time. There are a lot of things to do and a lot of room for experimentation. But it is simply unimaginable in Germany, it is just too far beyond the realm of possibility and the law. It is completely clear to me that it can never be done [at universities].” (German Community Member, Interviewee #1).

The Interviewee above seems concerned about the room they have as a university student. They argue that they would prefer more “room for experimentation”, e.g., in the form of science competitions, which are often offered to students as extracurricular activities.

As I pointed out earlier, Canadian DIY biologists are often concerned about the cost of higher education as an accessibility issue. However, with its largely free higher education system, German community members tended to pinpoint a lack of flexibility within research agendas and university curriculums as a core issue instead.

Like community biologists in the two other countries, German DIY biologists criticized that the university is too heavily regulated to act as an accessible epistemic space to its students or even interested publics.

Aspirations for a “space for experimentation” [German: Raum zum Experimentieren] are ever present in German DIY biologists’ imaginations of their movement. Among interviewees there seemed to be a general consensus that universities cannot offer the opportunities they are looking for. DIY biology spaces, tools and communities are thought to allow for a free flow of ideas and collaborations. “Space for experimentation” connotes not just visions of a physical space for experimentation but also of freedom and spontaneity. Moreover, experimentation, as a creative process, is envisioned as a necessity to innovate. Innovation is envisioned as a key to solve pressing social and political problems. This, again, invokes a sense of techno-optimism that is somewhat inherent to DIY biologists’ imaginings of their movement.

**Expert-public divide: Co-production and different publics:** German DIY biologists also share the vision of DIY biology as a challenge to the expert-publics divide. Again, this is thought to counter universities, which are thought of as inaccessible to publics:

“One of the advantages is that a community laboratory allows for horizontal knowledge co-production. It questions how knowledge production works at universities. Our movement wants to be inclusive; it wants to include different publics.” (German Community Member, Interviewee #8)

Interviews showed that German DIY biologists want to challenge the expert-public divide in two ways. First, they want to make alternative epistemic spaces accessible to individuals without university or industry affiliations. Second, they want to provide non-experts with educational opportunities.

German DIY biologists take a well thought-out, rather academic approach to how they envision to challenge the experts-publics divide. This is reflected in the ways in which they speak about publics and community laboratories. For instance, the above interviewee mentions “co-production” and “different publics”.

In a similar fashion, one interviewee voiced their distress about how they perceive non-experts to not be able to access scientific spaces in a meaningful way. Like the community member cited earlier, they had educated themselves about the history of public participation in science and have formed opinions on how publics should be involved. They voice that citizen science should offer more opportunities to engage in the sciences in meaningful ways. Thus, they are keen on creating alternative epistemic spaces, in the form of a DIY biology laboratory, which challenges the boundaries between experts/scientific institutions and publics:

“A 19^th^ century perception of the [non-expert] publics is a root cause in this context. Accordingly, a citizen may walk the illuminated halls of science once in a while, and somehow help out a bit. Count a couple of beetles and then be happy to have done some science too. At least that is what I have observed in Germany, and I think it is ridiculous. I would say that it just doesn’t live up to the ideal anymore. One should do it differently. One should open up a cool institute in Germany, where one can freely evolve. One could think differently.” (German Community Member, Interviewee #25)

This, and other interviewees, imagined DIY community laboratories to fill a gap. While they imagined universities and industry laboratories to remain largely inaccessible to publics, community laboratories were envisioned as an inviting forum for all.

Unlike in other country contexts, interviewees did not explicitly invoke the deficit model [71]. Instead, German DIY biologists seemed keen for non-experts to get involved in DIY biology without holding any expectations for how they should engage with, and perceive, science.

**Open-source biology and chaos computer club:** Open-source principles are more prevalent among German DIY biologists, as compared to their Canadian and British counterparts. A few of the members also report that they currently are, or at some point were, part of the open-source computer software community.

One contributing factor might be that several of my German DIY Biology community interviewees have, or once had, ties to Chaos Computer Club. Founded in 1981, Chaos Computer Club (CCC) is thought to be Europe’s largest association of hackers [72]. CCC is organized in regional hackerspaces and groups. The non-profit organization often weights in on policy discourses, specifically on technology security and privacy [73].

While describing their journey in open-source software, one interviewee excitedly described how they felt when first coming across DIY biology at a computer programming event:

“Open source has always been really important to me and when I heard that open-source bio exists I was like *wow* [emphasis added]” (German Community Member, Interviewee #9).

Many German community members report that open-source knowledge regimes are important to them and often a point of discussion at DIY biology community events. Open-source principles are thought to enable mutual aid and empowerment:

“One of the central themes, which is important to me, and that is ever-present in conversation with other community members, is that of community mutual aid and empowerment. The community helps each other out in any shape or form. This transfer of knowledge is shaped by open-source principles.” (German Community Member, Interviewee #8)

Accordingly, interviewees often gave detailed account of the ins and outs of sharing their knowledge with a wider audience. As I mention earlier, German DIY biologists place a lot of emphasis on organizing online. This also has an impact on how open-source principles are realized. Several members commented that forums and instant messaging groups have proven to be a useful tool in the open-source exchange of knowledge. They report that this exchange has become more effective with growing membership numbers on their various online platforms.

One DIY biologist reports that they thought that most, but not all, German DIY biologists are keen on open-source regimes. They recounted a project where they collaborated with other DIY biologists who wanted to commercialize the product they were developing:

“We were working on our [product], and they started to think that they’ll make a quick buck. So, they wanted to take over. And I told them that if they still want me on the team then it has to stay open source. I told them that they can go ahead without me, but I will definitely not support it. And then they started to berate me.“(German Community Member, Interviewee #9)

This quote again shows that open-source principles and a need to secure funding for DIY biology projects can at time be at odds. Moreover, it indicates that DIY biology communities’ envisioning of their movement, even within the same country, are not monolithic. My research finds that German DIY biologists imagine open-source principles to be tied to making findings/knowledge (e.g., protocols, blueprints) publicly accessible and thus reproducible or modifiable. Open-source ownership regimes do not necessarily mean that the resulting product is free (e.g., many DIY biology toolkits are produced and sold for a profit). However, the above quote illustrates that community members have their own interpretations, and thus visions of how, and if, open-source principles should be materialized. While open-source embodies ideas of openness, community-driven collaboration, and the free exchange of knowledge and resources, it is flexible term that is applied to a wide range of contexts, fostering innovation, accessibility, and cooperation in various fields.

#### 3.4.3. Intellectual freedom: Sustainability instead of profitability.

The third, and last, imaginary pertains to DIY biology fostering intellectual freedom. Like in Great Britain, this is the least dominant imaginary among German DIY biologists.

When DIY biologists invoke this imaginary, they primarily imagine intellectual freedom from institutional bureaucracy and research agendas. Unlike British DIY biologists, German DIY biologists did mostly not voice concern over institutional funding avenues.

Although German DIY biologists criticize academic research, their main critique is against capitalism, not the university. It seems that many German DIY biologists do not want to narrowly pursue commercial, for-profit endeavors but prioritise social relevance, specifically sustainability, over profitability.

What stood out in the case of German DIY biology were community members’ accounts of industry attempts to co-opt DIY biology initiatives. Several members talked about feeling uneasy about being approached by industry actors. It seems that industry sponsorships and DIY biology’s vision of intellectual freedom is difficult, maybe even impossible, to reconcile. This is how one community member explain how they encountered industry actors at an event with a DIY biology focus:

“They want to have everything, everything that they can get their hands on. They want to establish themselves in this area. I mean, what do management consultancies do? They parasitize. They want to know where the newest trends are and so on. It’s unpleasant. […] This is about venture capital, and this is also about tech start-ups and other start-ups not being enough for them. Do they now really have to exploit social initiatives as well?” (German Community Member, Interviewee #9).

This quote illustrates how this, like other German DIY biologists, imagine DIY biology as a sphere that allows for freedom from industry interests. Community members seemed concerned that commercial players have gained a presence at community events. If DIY biology can actualize intellectual freedom while being funded by industry, of course, is contested even among community members. This is to say that DIY biology’s relationship with industry is continuously negotiated and that there is not one clear stance on if, and under which circumstances, industry relationships can be reconciled with DIY biology’s vision of intellectual freedom.

#### 3.4.4. Socially relevant research agendas: A quest for sustainability.

Another prevalent imaginary among German DIY biologists is that of socially relevant research agendas.

Like DIY biologists in other countries, German DIY biologists view their movement’s potential against the backdrop of large-scale challenges such as the climate crisis. One organizer explains their goal when joining the movement as follows:

“The world is in flames, and I just want to help using the tools at my disposal” (German Community Member, Interviewee #9)

Interestingly, an overwhelming majority of German community members envisioned the movement’s social relevance to largely pertain to its potential to foster ecological change/transformation. Accordingly, numerous interviewees focus on sustainable agriculture and food systems, materials, and medicinal products. An especially large focus in German DIY biology is sustainable agriculture. For instance, DIY biologists are involved in sourcing and cultivating plants as low-carbon emission foods, meat-alternatives, or eco-friendly materials.

One contributing factor might be that German DIY biology community members tend to also be members of environmentalist groups. Their DIY biology activities and environmental activism often converge. Some DIY biologists explain to me that they came across DIY biology through one of these environmental initiatives. Interviewees also talk about collaborative efforts between sustainability initiatives and DIY biology’s groups, tools and methods. One community member praised how engaged DIY biologists are in matters of environmental and social concern:

“Community members are involved in numerous environmental projects. They are actively engaged in bringing about large-scale social and ecological transformation. I think it would be really great if there was a greater appreciation for [DIY biology’s] creative potential that is playing out this way.” (German Community Member, Interviewee #8)

Sometimes DIY biologists voice anti-institutional sentiments. In this context, it is noteworthy that community members talked about how they are also at times working with universities, among other organizations, to work on ecological transformation projects. Although German community members were critical of the academy, there seemed to be a consensus that universities are a fair partner for materializing socially relevant research agendas.

#### 3.4.5. Making German DIY bio: Space for experimentation.

German DIY biology materializes these imaginaries through concrete practices, spaces, and networks that reflect their values of democratization, intellectual freedom, and socially relevant research. The country’s community members focus on accessible, collaborative, and sustainable biological experimentation.

Much like their Canadian and British counterparts, German DIY biologists struggle with funding community spaces that meet their needs and comply with regulation. German DIY biologists found an alternative pathway by establishing a patchwork of community labs, pop-up workshops, and informal experimental setups that physically instantiate their imagined “space for experimentation.” These spaces often operate at the lowest biosafety level, reflecting both regulatory constraints and the community’s commitment to safety and responsibility. Rather than large, institutional-style laboratories, DIY bio spaces tend to be modest, flexible, and often temporary, i.e., hosted in co-working spaces, private basements, community centers, or shared maker spaces. This minimalist, low-cost infrastructure enables participation despite regulatory and financial barriers, thereby materializing accessible and open scientific environments.

Within these spaces, experimentation unfolds through hands-on projects that span from microbiology and plant biology to environmental monitoring and sustainable material development. Projects commonly focus on practical, socially relevant outcomes rather than on high-risk genetic modification, aligning with community members’ emphasis on safety, regulation adherence, and ecological sustainability.

Beyond physical labs, German DIY biology’s materialization includes extensive digital networks and open-source knowledge exchange platforms. Online forums, messaging groups, and repositories host protocols, project documentation, and collaborative discussions. These digital infrastructures reinforce the community’s commitment to openness and mutual aid, enabling knowledge to flow freely across geographical and disciplinary boundaries.

The strong influence of the Chaos Computer Club culture manifests in a shared ethos of transparency and peer-to-peer support. Community members report vibrant online and offline exchanges where expertise is shared, questions answered, and resources pooled, which indicates ways in which open-source ideals are practiced. These networks not only sustain day-to-day activities but also empower newcomers to join the movement, effectively lowering barriers to participation.

German DIY biology materializes its challenge to the expert-public divide by organizing inclusive events such as workshops, public lectures, citizen science projects, and hackathons. These activities serve as forums where non-experts and experts alike co-produce knowledge and engage directly with biological science. Community labs position themselves as accessible “entry points” to biology for diverse publics, embodying the imaginary of democratized science. By providing hands-on access to scientific tools and concepts, these events foster new publics who can meaningfully engage with science beyond traditional passive consumption.

The materialization of socially relevant research agendas is visible in the concrete projects pursued by German DIY biologists. Many community-led initiatives focus on sustainability challenges such as alternative food production, low-impact materials, and environmental monitoring. For example, community members cultivate plant-based proteins or develop bio-based packaging materials, integrating biological experimentation with environmental activism.

Collaboration with environmental NGOs and occasional partnerships with university researchers further embed DIY biology within a broader sustainability movement. These engagements materialize the community’s commitment to addressing the climate crisis and social ecological transformation, transcending purely scientific goals to include ethical and political dimensions.

Material realities of German DIY biology are deeply shaped by regulatory frameworks and community members’ safety-conscious practices. The community’s adherence to regulation and the precautionary principle reflects Germany’s risk-adverse approach to governing biotechnology. Compliance-related costs and complexity shape the scale and scope of material infrastructure, encouraging smaller, more nimble laboratories and activities.

Despite limited direct engagement with regulators, German DIY biologists express a strong interest in building relationships and clarifying regulatory grey areas. However, the materialization of these aspirations remains tentative, as uncertainty persists about whom to approach and through what channels. This contrasts with the Canadian and British contexts, where DIY communities are generally more aware of, and connected to, relevant regulatory points of contact.

## 4. Conclusions

When comparing imaginaries and smaller scale visions of DIY biology in Canada, Great Britain, and Germany, this paper shows how visions of extra-institutional science diverge and converge across different country-contexts. In my research, I almost exclusively encountered Canadian, British and German DIY biologists who hold qualifications in the natural sciences and most often are affiliated with traditional scientific institutions in industry or academia. It is particularly interesting that although they have access to research institutions, they still feel constrained and thus seek out alternative epistemic spaces.

DIY biology is thought to democratize biology by making research infrastructures more accessible, fostering open-source ownership regimes, and challenging the expert-publics divide. I find that these visions, in various nuances, are present in the imaginations of DIY biology communities in all three countries under investigation. At the heart of DIY biologists’ visions of democratizing biology is the goal of enrolling additional participants, which is then thought to foster innovation. DIY biologists’ perceptions of innovation are overwhelmingly positive. As I explain earlier, techno-optimism is inherent to DIY biology. In the countries under scrutiny, DIY biologists tend to exhibit a proclivity towards techno-optimism, resulting in a lack of critical examination of the assumption that science and technology innovation and entrepreneurship are inherently beneficial to society.

DIY biologists envisioning of their movement is entangled with academia-critical sentiments. Their critiques of academia slightly differ across country contexts but follow the same themes: Academia is perceived as inaccessible, overly bureaucratic and time consuming. Additionally, the academy is thought to fail at sufficiently addressing pressing problems of social relevance. In all three countries, academia is envisioned to “gatekeep” and slow down scientific exploration, while DIY biology is thought to democratize biology by making it accessible to publics independent of their background. Specifically Canadian DIY biologists tend to criticize the cost of higher education, which is thought to make biology inaccessible. Contrarily, German DIY biologists’ critique of the academy focuses on a perceived lack of flexibility in academic curricula and research agendas.

DIY biology is thought to democratize biology in various ways. For instance, open-source ownership regimes are envisioned to place collaborative production and learning over private (proprietary) ownership. DIY biologists imagine democratizing biology by making resources, such as educational tools and materials, available to publics. This again, is thought to help enroll additional participants, which is thought to foster innovation. Open-source ownership regimes are important to Canadian and German visions of the DIY biology movement but less prevalent in a British context.

In the context of the democratization of biology, Canadian and British DIY biologists tend to invoke the deficit model. DIY biologists envision that if non-experts had more and better access to biology, they would become more accepting of biotechnologies. Examples that are often mentioned in this context are genetically modified organisms (GMOs) or COVID-19 vaccines. As noted above, this line of argument invokes the deficit model [74] Community members claim that the interaction between them might enable laypeople to learn how to understand science like scientists, which is framed as the “correct” way [39].

Contrarily, evidence suggests that German DIY biologists do not invoke the deficit model when envisioning how they want to engage publics in their community spaces and projects. Instead, German DIY biologists take a considerate, reflective approach to how they envision challenging the expert-publics divide. My research found that German community members are rather keen on engaging publics in meaningful ways. Moreover, they manage their expectations of how they think that publics should perceive science, i.e., in their own unique ways and not necessarily like scientists.

Importantly, these imaginaries do not exist in isolation from practice. Across all three countries, the visions of democratized, accessible, and socially relevant science are shaped and constrained by material realities, including infrastructure, funding, and regulation. In Canada, materialization is marked by the tension between techno-optimistic individualism and the structural challenges of compliance and access. Canadian DIY biologists often draw on the garage entrepreneur imaginary, yet financial barriers and regulatory demands limit broader participation. While some entrepreneurial ventures such as Amino Labs embody attempts to lower barriers, the movement cannot quite overcome exclusions based on income, education, and access to resources.

In Great Britain, the materialization of DIY biology is similarly constrained by limited funding, overstretched volunteers, and a passive regulatory stance. British DIY biology communities struggle to enact their inclusive visions amid a lack of trained mentors and high entry barriers for complex experimentation. Despite efforts to position themselves as alternatives to institutional science, British DIY labs remain on the margins: recognized but not meaningfully integrated into national science policy or innovation ecosystems.

German DIY biologists, in contrast, materialize their visions through minimalist, flexible lab setups, extensive digital infrastructure, and socially oriented, sustainability-driven projects. Their focus on hands-on, low-risk experimentation and collaborative learning reflects a deliberate effort to align practice with values. German DIY biology exemplifies a materially grounded response to the challenges of access, participation, and responsibility. The influence of hacker culture and ecological activism is visible in how German communities design their spaces, organize events, and choose research priorities.

I want to note that across country contexts, in their envisioning of their movements, DIY biologists sometimes forget to acknowledge the privilege associated with pursuing science research and education. Specifically, among Canadian DIY biologist, DIY biology is not only imagined as a “better way forward” but also as a means to getting back to how “science used to be”. This is to say that some tend to think of DIY biology as a movement that incorporates aspects of 17th century European gentleman science. DIY biology is thought to let individuals pursue scientific methods outside of academia, as gentleman scientists are thought to have done. Thereby, DIY biology is imagined as democratizing research by making its practices and spaces more accessible. This is envisioned to remedy grievances associated with traditional research institutions, such as acquiring funding and publishing research papers. However, up until the mid-20^th^ century scientific research was a privilege almost exclusively reserved to affluent, privileged, white male individuals. This is at odds with DIY biologists’ vision of democratizing biology. One could argue that gentleman science can be thought of as even more exclusionary than neoliberal academia. Across different country contexts, some DIY biologists do not seem to have a nuanced understanding of which demographics can get involved in science. DIY biologists in all three countries are often highly educated individuals that are able to use their own funds to pursue extra-institutional science. That in itself is a privilege that is not often explicitly acknowledged among DIY biology community members.

The second imaginary at the core of DIY biology is that of intellectual freedom. Out of all three countries under investigation, this imaginary is most often invoked in the Canadian context. Canadian DIY biologists envision that alternative epistemic spaces offer a curiosity-driven approach, and more independence and agency as compared to academia or industry. Canadian community members seem to place a lot of value on individual self-determination and personal development. This is to say that the legend of the garage entrepreneur places a lot of value on individualism, evoking “the image of the lone individual who relies primarily on his or her extraordinary efforts and talent” [75, p. 2].

In Great Britain and Germany, the imaginary of DIY biology facilitating intellectual freedom is the least prevalent of the three imaginaries. When British DIY biologists invoke this imaginary, they imagine intellectual freedom along two lines: Freedom from institutional bureaucracy and academic/industrial research agendas and freedom from the interests and ties that come with traditional funding avenues (i.e., academic grants or corporate sponsorships). Similarly, when German DIY biologists invoke this imaginary, they also imagine intellectual freedom from institutional bureaucracy and research agendas.

German DIY biologists mainly voice their critique against capitalism, not the academy. What stood out in the case of German DIY biology are community members’ concerns with industry attempts to co-opt DIY biology. It seems German DIY biologists find it difficult, if not impossible, to reconcile industry sponsorships and DIY biology’s vision of intellectual freedom. This is to say that specifically German DIY biology’s relationship with industry is continuously negotiated and there is not one clear stance on if, and under which circumstances, industry relationships can be reconciled with visions of intellectual freedom.

Moreover, compared to their Canadian counterparts, British and German DIY biologists less often imagine DIY biology as an avenue for personal development. Instead, they more often envision collective benefit, e.g., community projects with social relevance. Specifically British DIY biologists’ visions of their movement focus on what can be collectively gained rather than individual freedoms. For instance, British DIY biologists place a lot of emphasis on working with scientists in the Global South to improve scientific infrastructures.

This is connected to the movement’s third major imaginary, which is that DIY biology has the potential to tackle socially relevant research topics. DIY biologists often claim that traditional research institutions fail to address areas of so-called undone science as they are thought of as too bureaucratic and/or monetarily motivated [50,51].

It should be noted that some, but not all, Canadian DIY biologists maintain that their movement can effectively address socially relevant research agendas. In fact, some Canadian movement members point out that communities do not have the capacities to address socially relevant research agendas as well, or even more effectively, than research institutions.

Yet, German DIY biology appears more consistent in aligning this imaginary with its practices. The environmental orientation of many German projects, which are focused on sustainability, e.g., alternative food production, or biodegradable materials, demonstrates a commitment to integrating biological experimentation with activism. These projects reflect a vision of science not just as knowledge production, but as an instrument for ecological and social transformation.

This paper examined how DIY biologists in Canada, Great Britain, and Germany imagine their movement, highlighting the interplay between global sociotechnical imaginaries and country-specific socio-cultural and political contexts. By empirically investigating the country-specific manifestations of shared imaginaries — democratization of science, intellectual freedom, and the pursuit of socially relevant research — this study contributes to the growing literature on grassroots innovation and novel technology movements [2,76–78]. It emphasizes that while DIY biology is internet-native and shaped by globally shared ideals, its practices and community dynamics are deeply rooted in local realities.

Ultimately, understanding these imaginaries in relation to their material enactments reveals the uneven ways in which DIY biology communities succeed, or struggle, to realize their visions. Material constraints do not simply limit these visions; they shape and sometimes transform them. The tensions between aspiration and implementation illuminate the practical politics of grassroots science and the infrastructural conditions necessary to sustain it.

While this paper focuses on imaginaries, it is important to note that these visions are enacted in practice by DIY biologists as users of tools, infrastructures, and technologies. They are not passive adopters but actively adapt, reconfigure, and reshape their research environments. DIY, making, hacking, and tinkering deserve more complementary perspectives to better understand how globally shared imaginaries are realized through localized practice.

Looking ahead, this research opens pathways for exploring how other countries engage with DIY biology and whether different regulatory or cultural conditions produce distinct imaginaries. Furthermore, as debates around biosecurity, ethics, and inclusivity in science continue to evolve, future studies could investigate the role of DIY biology in challenging or reinforcing traditional boundaries in science and technology. This work underscores the importance of a culturally sensitive approach to understanding grassroots movements in the life sciences, providing a framework for analyzing the broader social impacts of emerging biotechnologies.
